# Super-Oxygenation in the Dynamics of Organic Life

**Published:** 1869-07-15

**Authors:** Z. C. M’Elroy

**Affiliations:** Zanesville, Ohio, President Muskingum County Medical Society


### COMMUNICATIONS.

### *Super-Oxygenation in the Dynamics of Organic Life.*

BY Z. C. M'ELROY, M.D., ZANESVILLE, OHIO,

President Muskingum County Medical Society.

The reply of your correspondent, S. S. Wallihan, M.D., Chicago, to "Criticism and Strictures on Super-oxygenation," must be taken as evidence that human nature has not materially changed in eighteen hundred years. Iconoclasts and Fetich existed then, as they exist now. The *animus* of both appears to be substantially unchanged, as shown by the following bit of history: "And the same time there arose no small stir about that way; for a certain man named Demetrius, a silversmith who made silver shrines for Diana, brought no small gain to the craftsmen, whom he called together with the workmen of like occupation, and said, Sirs, ye know by this craft we have our wealth; moreover, ye see and hear, that not alone at Ephesus, but throughout all Asia, this Paul hath persuaded and turned away much people, saying that they be no gods, which are made with hands, so that not only this our craft is in danger to be set at nought, but, also, that the temple of the great goddess Diana should be despised, and her magnificence should be destroyed, whom all Asia and the world worshipeth. And when they heard these sayings, they were full of wrath, and cried out, saying, Great is Diana of the Ephesians." (1)

Dr. Wallihan, like the Ephesian craftsmen, seems to have been filled "full of wrath by my accidental strictures and criticism" of his published cases, healed by super-oxygenation, and consequently, both dignity and courtesy are absent from his reply, which may be summed up in these words, "Great is super-oxygenation in the treatment of disease, especially when inhaled through one of 'my machines.'" (2)

From the whole temper and nature of his reply, Dr. W. evidently sees in Dr. Zeigler what he esteems to be a "fellow craftsman," for whom he only has sweetness and light; but, in myself he beholds an iconoclast, upon whom he proceeds to pour out his wrath in vulgar personal abuse.

Experience, clinically, is the court of last resort in all matters therapeutical, and the decisions there rendered are final and conclusive. Super-oxygenation, through Dr. Wallihan's "machine," has yet to pass this ordeal, and what the result may be it is not difficult to predict. Apparently, it would be in favor of the utility of direct super-oxygenation only in certain cases, though not to the exclusion of super-oxgenation by means of chlorate, or permanganate potassa, aqua regia, and other substances containing large volumes of oxygen in unstable chemical combinations, nor to the abandonment of the increased frequency and volume of oxygen introdued into the system during physical exertion in the open air of the country, and divers other therapeutical measures, all having for their end an increase in the velocity of the organic processes of nutrition and oxida-

---

(1) Acts xix: 24–28.

(2) See adv. Drs. Wallihan & Wilford, Chicago Medical Journal, June 15, 1869.

tion, a disarrangement of which occasions all states of the system included under the generic idea of disease.

No doubt exists in my mind as to the gradual, not to say more rapid, growth in the professional mind of simpler and truer views of life, as well as of disease and its therapeutical management, than is taught in our schools, or contained in text-books, for they can hardly be expected to represent more than the average status of the professional mind of the age. Text-books, as a rule, follow, but do not lead in medical progress. This crops out in monography, journals, etc., etc., before being adopted in text-books, or taught in the schools.

This is evidenced in many ways. Thus, the fact that the only impenetrable mystery of life is its *forms*, is the lesson of Prof. Huxley's recent lecture on *the physical basis of life.* Another is the more general recognition of the fact that the human body is composed of ordinary elements, widely distributed through nature; and its organization controlled, except in its forms, by the ordinary forces of inorganic nature. As over forms, therapeutic agents exercise no control whatever, they must be left out of therapeutical discussions. And as forms are the only feature of the human body unaccounted for by the operations of ordinary modes of the physical forces, it is to these that the term vital is alone applicable, and as over these therapeutics, have no control. The *vital force*, or preserver of forms amidst the incessant molecular changes of organized life, can no longer embarrass therapeutical discussions. The idea of a something in life, represented by the word *vital*, beyond comprehension, will no longer repel investigation, or serve as a cover for ignorance. The human body, physiologically, is very aptly illustrated by a burning candle, which gives light and heat at the expense of its substance, by combustion, *i. e.*, the carbon and hydrogen of the candle, combining with oxygen of the surrounding atmosphere; the result is light, heat, carbonic acid and water. So, in organic dynamics, force, in any of its forms, can only be manifested at the expense of the tissues of the body. The food (protoplasm) is mainly, if not wholly, converted into organized tissues or textures, before oxidation and the production of force, *i. e.*, heat, mechanical motion, sensation, emotion, intellection and psychological phenomena.

And once again, the meaning of the *restorative system of medicine*, is, that man is superior to his maladies; and that disease is something less, not something added to life. Finally, the recognition of the unity of the body in its healthy, as well as disarranged conditions in disease, is an indispensable requisite to the free development of physiology, pathology and therapeutics.

These are pretty certain to form part of the creed of the coming physician, quite as certain as that coming events cast their shadows before them.

In conclusion, what oxygen—free oxygen—does in the laboratory of organic life, cannot be mistaken. The eighteen inspirations per minute in health, and greater frequency in disease, which are necessary to the continuation of life; the disturbances which instantly occur from an interruption of the almost continuous introduction into the system of free oxygen at the lungs, or change in its proportion to the atmosphere, or the introduction into it of other gases than those nominally contained in it, and the narrow range of temperature of the body in health, and its constantly accompanying changes in diseased conditions, are conclusive as to what oxygen does in organic life. Introduced into the system, if it does not combine with anything, it is a negation; if it combine with anything force will be liberated, and beyond the normal requirements of the system for force, it will appear as free heat. Free oxygen, introduced at the lungs, has, so far as science has determined, but one mission, and that is reduction, not building up, only as a secondary result of the liberation of force.

